# Loss of Seizure Control in a Patient With Vitamin D Deficiency and Phenytoin-Induced Hypocalcemia

**DOI:** 10.7759/cureus.32407

**Published:** 2022-12-11

**Authors:** Tiago Branco, Ana Cardoso, Ana Baltazar, Catarina Gonçalves, Vera C Santos

**Affiliations:** 1 Internal Medicine, Hospital de Santa Maria, Centro Hospitalar Universitário Lisboa Norte, Lisboa, PRT; 2 Intensive Care Medicine, Hospital Professor Doutor Fernando Fonseca, Amadora, PRT

**Keywords:** calcium and vitamin d, anti epileptic drugs, loss of seizure control, phenytoin induced hypocalcemia, phenytoin

## Abstract

Phenytoin is a widely used antiseizure drug with well-documented side effects, including hypocalcemia, particularly in patients with concomitant vitamin D deficiency. Decreased serum calcium levels can induce seizures. In stabilized patients under long-term anticonvulsant treatment with phenytoin, loss of seizure control is rare but has been reported. This report illustrates a case of a 69-year-old woman under treatment with phenytoin for more than 10 years, who presented persistent hypocalcemia despite calcium correction, and seizures refractory to treatment with four combined antiepileptic drugs. She also presented with low vitamin D and elevated parathyroid hormone levels. Only when phenytoin administration was stopped it was possible to correct hypocalcemia and achieve seizure control. This case illustrates the need for regular monitoring and supplementation with calcium and vitamin D for patients under prolonged treatment with phenytoin. The proposed mechanism for phenytoin-induced hypocalcemia is reviewed. When installed, hypocalcemia can be resistant to supplementation until phenytoin is stopped, and in rare cases may lead to loss of seizure control.

## Introduction

Hypocalcemia is a well-known effect of many antiseizure drugs. Phenytoin is a widely used antiseizure drug, which blocks voltage-dependent neuronal sodium channels [1], with well-documented side effects (cardiovascular, central nervous system related, dermatologic, endocrinal, and musculoskeletal). It has been associated with decreased serum calcium (Ca) levels and bone metabolism disorders both in patients with normal and low vitamin D Levels [2,3].

Decreased serum Ca levels can induce seizures. An inverse relationship between extracellular Ca and neuronal excitability has been specified as the mechanism behind this phenomenon [4]. Despite being an antiseizure drug, phenytoin-induced hypocalcemia can also induce loss of seizure control in patients undergoing long-term treatment [5]. Nevertheless, this effect in stabilized patients is rare [6]. Toxic doses of phenytoin (superior to 20 ug/mL) have also been reported to cause seizures. 

## Case presentation

**Figure 1:**
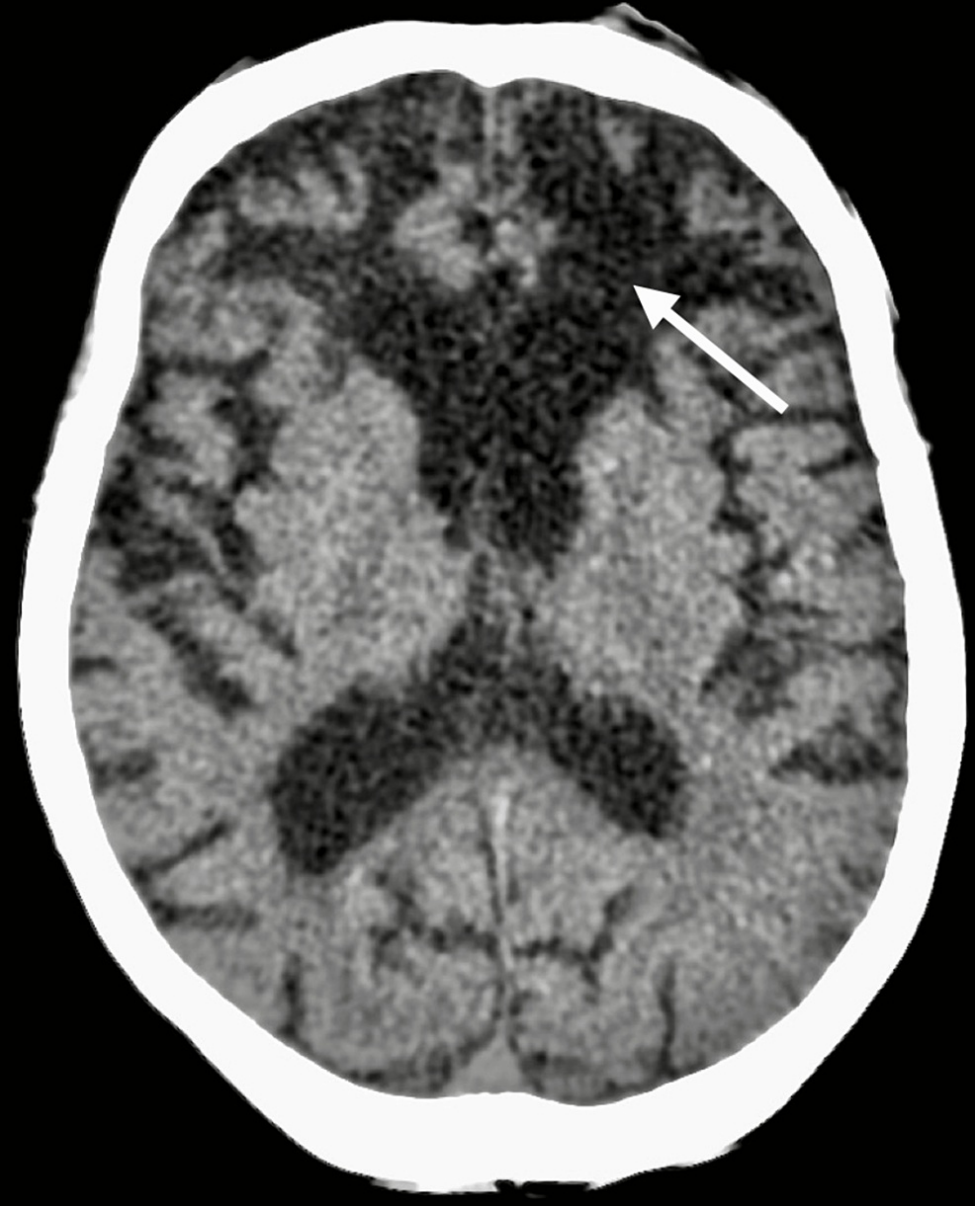
CT scan showing old contusion trauma sequels and a pattern of cortical-subcortical atrophy

**Figure 2:**
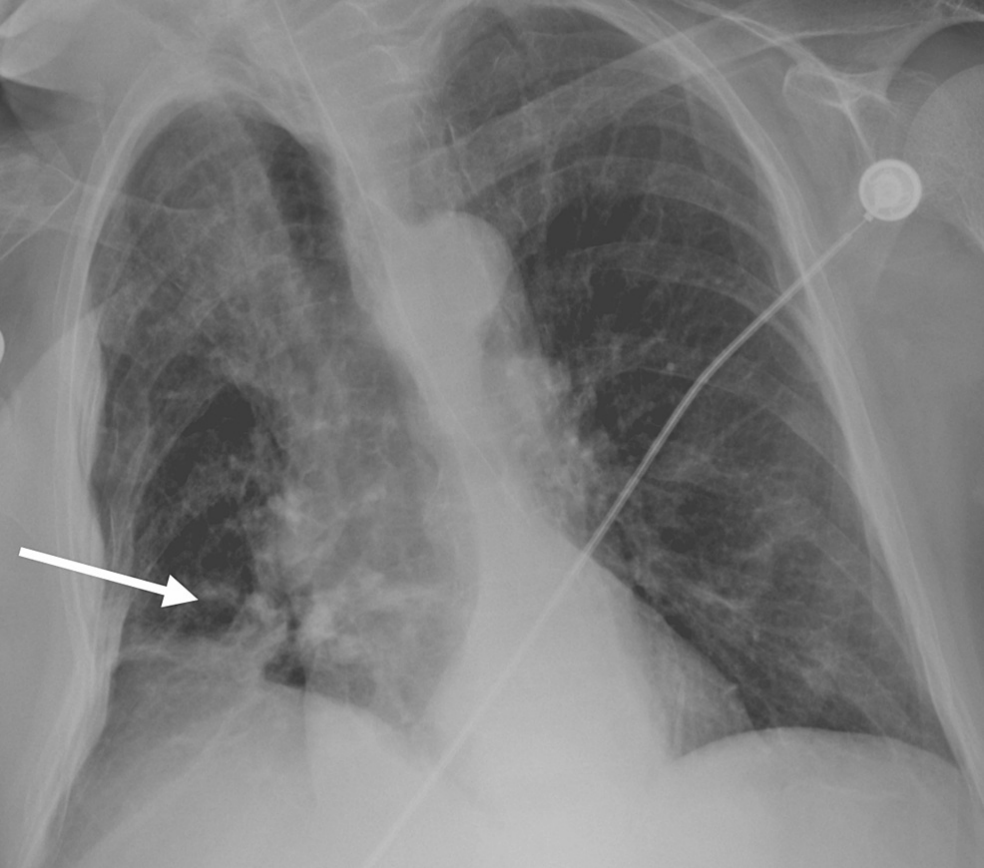
Chest x-ray documenting aspiration pneumonia

A 69-year-old woman with a previous history of epilepsy, alcohol abuse, and neurocognitive disorder was admitted to the emergency department (ED) for altered mental status. According to her husband, she had been experiencing increasingly frequent seizures for the last few months, despite regular medication with phenytoin (100 mg twice daily), and valproate (500 mg daily). In the three days prior to the admission, her state was described as comatose. On examination at the ED she was afebrile, non-responsive, and presented with elevated creatine kinase (CK) (1683 U/L; normal range 30-145 U/L), and elevated WBC count (12.11x10^9/L; normal range 4.5-11x10^9/L) with elevated C-reactive protein (CRP) (11.2 mg/dL; normal < 0.9 mg/dL). No signs of alcoholic intoxication were detected. Serum and ionized calcium were low (5,5 mg/dL; normal range 8.5-10.2 mg/dL and 0.61 mmol/L; normal range 1.20-1.32 mol/L, respectively), valproate levels were infra-therapeutic (2,8 ug/mL; normal range 50-100 ug/mL) and phenytoin levels were therapeutic (11.6 ug/mL; normal range 10-20 ug/mL). Head CT scan (Figure 1) revealed old contusion trauma sequels and a pattern of cortical-subcortical atrophy, and chest X-ray revealed condensation areas on the right lung (Figure 2).

Aspiration pneumonia was assumed in the context of a previous seizure episode, and empiric antimicrobial therapy with amoxicillin-clavulanate (2000 mg/125 mg twice daily for seven days) was started on admission to the medical ward. 

On the following day, she presented a generalized tonic-clonic seizure. Electroencephalogram documented a convulsive status epilepticus, and levetiracetam and clonazepam were added to the previous phenytoin and valproate scheme. Further investigation to exclude other causes of persistent hypocalcemia revealed low Vitamin D (3 ng/mL), elevated parathyroid hormone (PTH) (242 pg/mL), and low urinary Ca (4.2 mg/dL). 

Despite therapeutic levels of four antiepileptic drugs, with calcitriol and intravenous Ca correction for 48 hours, new seizures were witnessed, and serum Ca levels remained persistently low (5.6 mg/dL). Phenytoin administration was then stopped. After this therapeutic change, a gradual correction of serum Ca to normal levels was achieved and subsequent electroencephalograms revealed no more epileptic activity. The patient gradually regained consciousness and recovered to her basal status. 

After discharge, in follow-up appointments, she was able to withdraw valproate and remained controlled with no further seizures under levetiracetam (1500 mg twice daily), clonazepam (0,5 mg twice daily), and supplementation with oral cholecalciferol and calcium. 

## Discussion

Low serum Ca levels stimulate parathyroid glands to increase the production of parathyroid hormone (PTH). Elevated PTH levels promote bone osteoclastic resorption of Ca and inorganic phosphate (Pi). In the kidney, PTH increases the reabsorption of Ca and decreases the reabsorption of Pi [2]. Additionally, dietary (or supplementary) vitamin D is metabolized in the liver to 25-OH-cholecalciferol (under cytochrome P450 regulation). In the kidney, PTH promotes the transformation of 25-OH-cholecalciferol into its active metabolite 1.25-OH-cholecalciferol, which after binding to intestinal steroid hormone receptors and a Ca-binding protein can increase absorption of dietary Ca into the enterocytes [3].

The proposed mechanism for phenytoin’s impairment of Ca absorption is twofold. On one hand, it presents a dose-dependent inhibition of the active Ca transporters in the intestinal parietal cells. However, phenytoin also acts in the kidney as an agonist of the catabolic enzyme that cleaves 25-OH-cholecalciferol and 1.25-OH-cholecalciferol into their inactive metabolites [7,8].

This loop mechanism explains how long-term use of phenytoin can result in seizure-inducing hypocalcemia even in the presence of Ca and vitamin D oral supplementation. However, this model fails to explain why, in the case of our patient, intravenous Ca gluconate administration was not effective for correcting hypocalcemia until phenytoin administration was interrupted.

## Conclusions

Long-term treatment with phenytoin can induce hypocalcemia, which in rare cases may lead to loss of seizure control. In patients under prolonged treatment with phenytoin, monitoring and supplementation with Ca and vitamin D should be conducted regularly. Bone density studies should also be regularly performed. When installed, hypocalcemia can be resistant to supplementation until phenytoin is stopped. 
